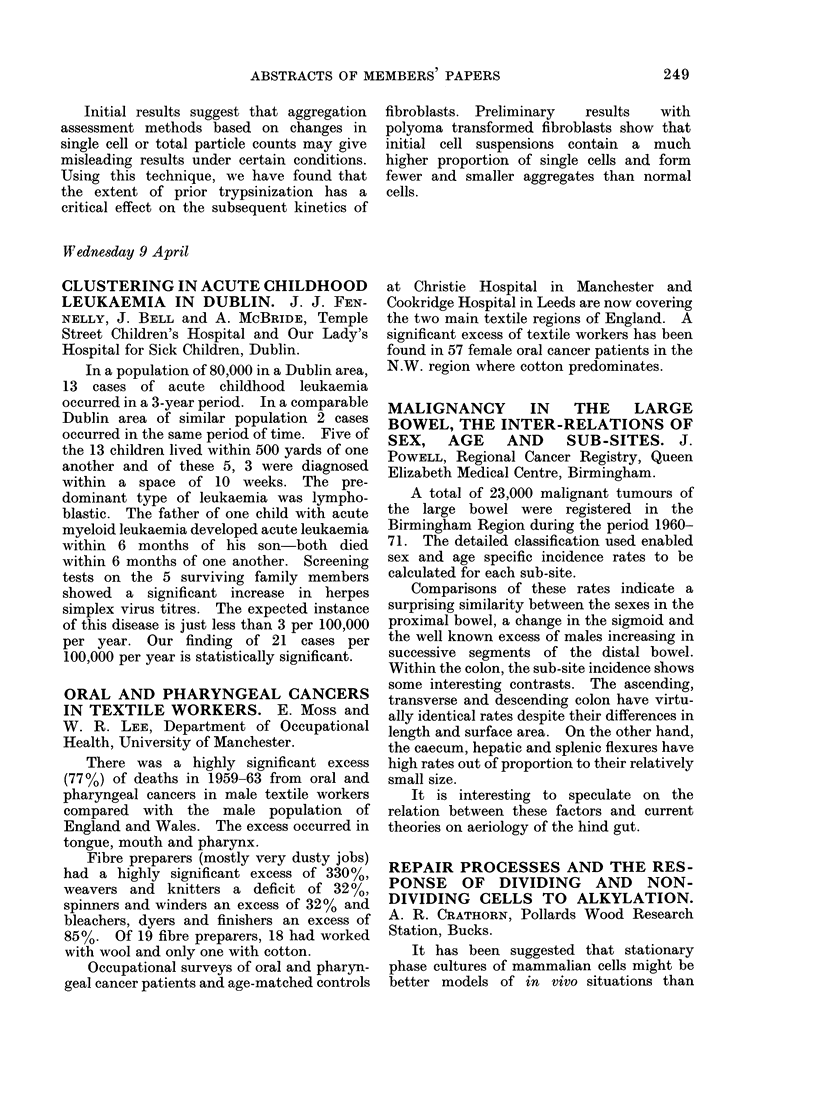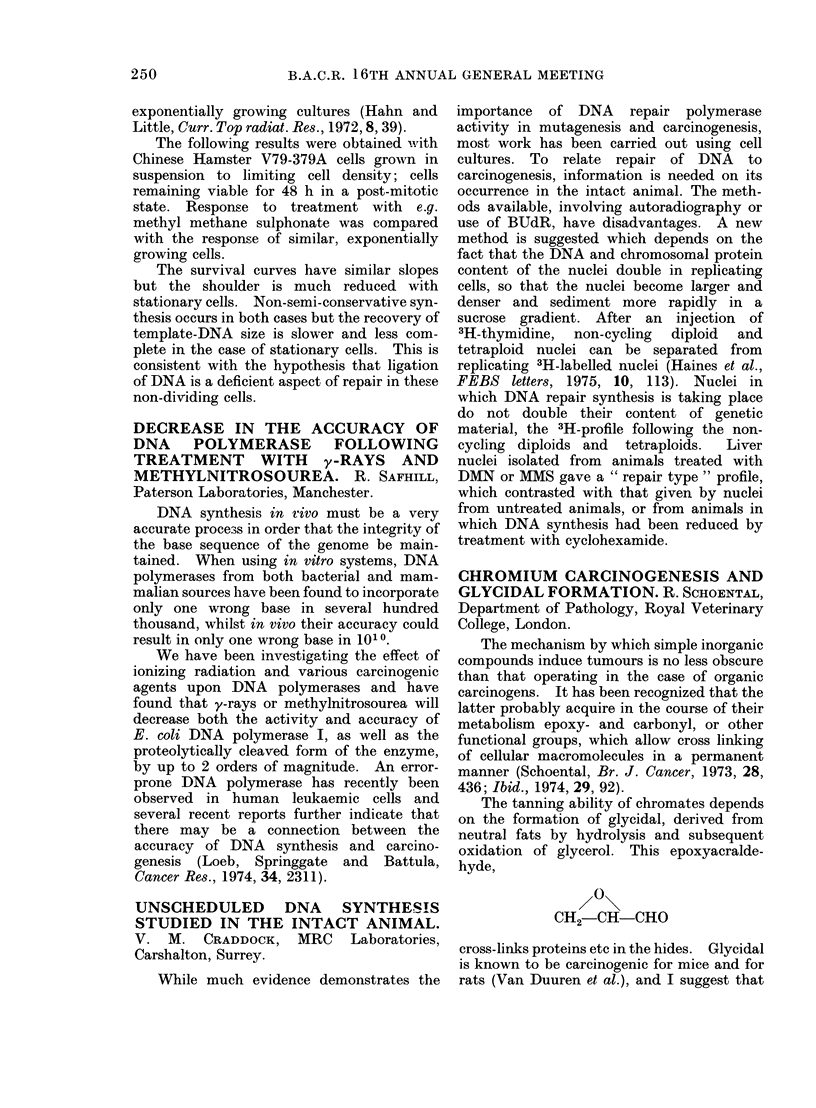# Proceedings: Repair processes and the response of dividing and non-dividing cells to alkylation.

**DOI:** 10.1038/bjc.1975.187

**Published:** 1975-08

**Authors:** A. R. Crathorn


### REPAIR PROCESSES AND THE RESPONSE OF DIVIDING AND NON-DIVIDING CELLS TO ALKYLATION.

A. R. CRATHORN, Pollards Wood Research Station, Bucks.

It has been suggested that stationary phase cultures of mammalian cells might be better models of *in vivo* situations than exponentially growing cultures (Hahn and Little, *Curr. Top radiat. Res.*, 1972, **8**, 39).

The following results were obtained with Chinese Hamster V79-379A cells grown in suspension to limiting cell density; cells remaining viable for 48 h in a post-mitotic state. Response to treatment with *e.g.* methyl methane sulphonate was compared with the response of similar, exponentially growing cells.

The survival curves have similar slopes but the shoulder is much reduced with stationary cells. Non-semi-conservative synthesis occurs in both cases but the recovery of template-DNA size is slower and less complete in the case of stationary cells. This is consistent with the hypothesis that ligation of DNA is a deficient aspect of repair in these non-dividing cells.